# Compulsory Vaccination

**Published:** 1886-01

**Authors:** 


### COMPULSORY VACCINATION.

The subject of compulsory vaccination as a safeguard against small-pox as, indeed, the practical advantages of vaccination over its disadvantages to the individual, has, in any case, given rise to so much controversy in this country, that it is deemed that some extracts from the minutes of a discussion of the same questions, by the Parisian "Academy of Medicine" over the report upon the service of vaccinations in France during the year 1884, presented to the Academy by M. Blot, in the name of the Permanent Commission of Vaccination and Retro-Vaccination, of that country, will be of interest to our readers. We are indebted to a late issue of the *Journal d'Hygiene* for what is given.

M. Blot, in the outset, had taken ground against the anti-vaccinationists, and had also shown a certain predeliction for re-vaccination, and more than once affirmed the necessity of petitioning parliament to enact a law which should impose upon all persons the duty of vaccination as a preventive measure. Upon the first point M. Guerin observed that the opponents of vaccination were neither fools nor lunatics, neither do they, in the language of the Academy, unite in themselves any such qualities.

Upon the third point, the learned president stated that the minority of the Academy who of posed compulsory vaccination, towards the close of debate were strong enough to show that, in making such legal rules concerning the admission of children into the public schools, and of persons into the civil and military service, as this measure would impose, would necessitate a system of legal proscription more or less systematic and invidious.

Upon the second point which we have reserved for the close, in order to develop it more fully, M. Blot had to undergo a cross-fire of doubts and objections presented with more or less energy, by M. M. Bucynoy, Vidal, Maurice, Perrin and Hardy.

M. Bucynoy reminded his conferrers that this same question, so recently raised by the Medical Society of the Hospitals, had given place to a discussion which demonstrated that vaccin had, in a great measure, lost its efficacy.

M. Vidal argued with his collegues of the hospitals as to the insufficiency of vaccinations performed by the aid of vaccine gathered from an adult after *re*-vaccination, stating that he had had occasion to vaccinate a person on whom the innoculation produced perfect pustules, and three months afterwards he re-vaccinated the same person with like vaccin successfully.

M. Perrin said that recently, when the army Sanitary Committee, in providing medical stores, insisted upon the utility of re-vaccination, he was very careful to observe that adulterated vaccin had been used to such an extent that it was almost impossible to procure *pure vaccin*, either from animals or healthy young women.

M. Hardy was, as usual, opposed to *half* terms either the vaccin, obtained from re-vaccinations is *good* or it is *bad*. If it is good it will serve its purpose, but if found to be bad or uncertain I agree with the Academy, that it should never be used, for the effect of its employment is to give a *false security* at all times dangerous because it leads one to neglect the truly efficacious means of prev ention.

The President, in closing the discussion, reviewed the various opinions which had been offered, and after complimenting the views of M. M. Hardy, Bucynoy, Vidal, et sec., remarked that the divers statements showed that some preparations of vaccine were less reliable than others ; that the prescriptions which are recommended by the Academy have the example of Belgium of Italy and other countries.

He expressed himself as in favor of the utility of vaccination, as a preventive, but the vaccin employed should be purified by successive cultures, after which it could be prescribed with entire confidence in its successful operation.

This much for the views some of the members of the Parisian Academy of Medicine, which, as a body, favors vaccination.

The recent alarming spread of small-pox in Montreal, even to the partial prostration of business and the stubborn opposition to vaccination on the part of large numbers of persons whose resistance cost some of them their liberty, under the rule of compulsory vaccination, are things of too recent occurrence to be out of mind, and certainly add gravity to a subject upon which wide differences of opinion exist in this country.

---

M. Fayol has ascertained that the absorption atmospheric oxygen by coal dust usually produces the rise in temperature to which spontaneous combustion is due. Lignite is ignited at the low temperature of 300 deg., anthracite at 575 deg., and other varieties of coal in a powdered state, at intermediate temperatures.

